# Bioinformatics Analysis of WRKY Family Genes in Flax (*Linum usitatissimum*)

**DOI:** 10.3390/life13061258

**Published:** 2023-05-25

**Authors:** Xia An, Qin Liu, Hui Jiang, Guoyun Dong, Danqing Tian, Xiahong Luo, Changli Chen, Wenlue Li, Tingting Liu, Lina Zou, Jinyao Ying, Huaping Zhou, Xuan Zhu, Xiaoyan Chen

**Affiliations:** 1 Zhejiang Xiaoshan Institute of Cotton & Bast Fiber Crops, Zhejiang Institute of Landscape Plants and Flowers, Zhejiang Academy of Agricultural Sciences, Hangzhou 311251, China; lq18388746921@163.com (Q.L.); tdqing@zaas.ac.cn (D.T.); luoxh@zaas.ac.cn (X.L.); chenchangli@zaas.ac.cn (C.C.); liwenlue@zaas.ac.cn (W.L.); liutt@zaas.ac.cn (T.L.); zoulina@zaas.ac.cn (L.Z.); 2 Henan Academy of Agricultural Sciences, Zhengzhou 450002, China; 12jiang31hui@163.com; 3 Zhangjiajie Research Institute of Agricultural Science and Technology, Zhangjiajie 427000, China; daledgyun@163.com; 4 Hangzhou Xiaoshan District Agricultural (Forestry) Technology Promotion, Hangzhou 311203, China; xsnyyjy19651@163.com (J.Y.); zhou_huaping@163.com (H.Z.); 5 Dali Bai Autonomous Prefecture Agricultural Science Extension Research Institute, Dali 671699, China; zhuxuan2006@163.com (X.Z.); dalichenxiaoyan@163.com (X.C.)

**Keywords:** flax, *WRKY*, transcription factor, gene family

## Abstract

*WRKY* gene family is one of the largest transcription factor families involved in various physiological processes of plants. Flax (*Linum usitatissimum*) is an important stem fiber crop, and it is also an economically important crop in natural fiber and textile industries around the world. In this study, 105 *WRKY* genes were obtained by screening the whole genome of flax. There were 26 in group I, 68 in group II, 8 in group III and 3 in group UN. The characteristics of the WRKY motif and gene structure in each group are similar. The promoter sequence of *WRKY* genes includes photoresponsive elements, core regulatory elements and 12 cis-acting elements under abiotic stress. Similar to *A. thaliana* and *Compositae* plants, *WRKY* genes are evenly distributed on each chromosome, with segmental and tandem repeated events, which play a major role in the evolution of *WRKY* genes. The flax *WRKY* gene family is mainly concentrated in group I and group II. This study is mainly based on genome-wide information to classify and analyze the flax *WRKY* gene family, laying a foundation for further understanding the role of WRKY transcription factors in species evolution and functional analysis.

## 1. Introduction

WRKY transcription factor is one of the largest transcription factor families at present [1]. As a positive and negative regulator of plant defense regulation and abiotic stress [2], it plays an important role in plant growth, development, senescence and biotic and abiotic stress processes [1,3,4,5]. WRKY*s* play an important role in the regulation of senescence under biotic stress and participate in the differentiation of flowers and buds and the development of lateral roots and trichomes [6]. WRKY transcription factors are regulated by hormones such as salicylic acid (SA), abscisic acid (ABA) and gibberellin (GA) under biotic and abiotic stresses and play an important role in many physiological processes through the regulation of hormone signals [2]. In order to cope with different biotic stresses, the transcription factors change the transcription levels and protein post-processing of related genes by activating signal pathways such as SA, jasmonic acid (JA), ethylene (EI) and other pathways, thus realizing the biotic stress of functional plants [7]. WRKY transcription factors can participate in a variety of plant hormone signaling pathways and regulate other physiological processes, including fruit ripening and leaf senescence [1]. For example, *WRKY75* is a positive regulator of leaf senescence, and the loss of its function delays leaf senescence [1], while At*WRKY70* is a negative regulator of developmental senescence [2]. *Arabidopsis thaliana* defense-related transcription factors *WRKY54* and *WRKY70* can regulate gene expression mediated by SA and can be used as negative regulatory factors of SA synthesis, which can enhance the tolerance to osmotic stress by regulating stomata closure [8]. *HaWRKY10* can reduce carbohydrate metabolism of sunflower through the ABA/GA pathway and gluconeogenesis, so as to improve seed lipid metabolism [9]. *AtWRKY18*, *AtWRKY40* and *AtWRKY60* not only play a role in ABA response, but also play other important roles in *A. thaliana* [10]. Overexpression of *OsWRKY11*, *OsWRKY71*, *OsWRKY72* and *OsWRKY77* in rice can be induced by ABA, and the expression of *OsWRKY24* and *OsWRKY45* can be reduced or negatively regulated [11]. *OsWRKY08* can improve the osmotic stress tolerance of transgenic *A. thaliana* by positively regulating the expression of ABA response genes [12]. Transgenic rice plants with *OsWRKY45* and *OsWRKY72* genes can improve their tolerance to drought and salt stress [7]. *OsWRKY58* plays an important role in promoting seed development and stem elongation in response to salt stress [13]. Rice plants with *OsWRKY80* have strong resistance to rice blast, and *OsWRKY80* and *OsWRKY4* play a positive regulatory loop role in disease resistance [14]. *GmWRKY13*, *GmWRKY21* and *GmWRKY54* in soybean play different roles in abiotic stress. *GmWRKY13* plays a role in the development and stress of lateral roots, *GmWRKY21* transgenic *A. thaliana* plants showed high cold tolerance and *GmWRKY54* showed strong salt tolerance and drought tolerance [15]. Overexpression of *ZmWRKY106* has been shown to improve drought tolerance of *A. thaliana* [16]. Transgenic wheat plants with *TaWRKY2* and *TaWRKY19* genes have high tolerance to drought stress [12]. Overexpression of *TaWRKY146* in wheat showed sensitivity to salt and drought stress, while *FtWRKY46* in tartary buckwheat (*Fagopyrum tataricum* (L.) Gaertn) showed increased tolerance to salt stress by scavenging reactive oxygen species (ROS) [11]. Overexpression of *HvWRKY6* in transgenic barley (*Hordeum vulgare* L.) and *HvWRKY70* in transgenic wheat (*Triticum aestivum* L.) plants can increase resistance to stripe rust [11]. Overexpression of *AtWRKY21*, *AtWRKY33*, *AtWRKY40*, *AtWRKY57* and *AtWRKY70* in *A. thaliana* improved stress tolerance in an ABA-dependent manner [11]. *AtWRKY18*, *AtWRKY40* and *AtWRKY60* belong to the basic defense and they are negative regulatory factors with additive and antagonistic effects [14]. Overexpression of *AtWRKY75* accelerated the senescence of leaves. *AtWRKY23* is involved in the development of plant roots by controlling the distribution of auxin (IAA) [14]. *AtWRKY23* regulates the production of flavonols through the induction of auxin, and its metabolites give negative feedback on the signal transduction of plant hormones [17]. *AtWRKY28* and *AtWRKY75* may play a role through the JA/ET pathway [18]. *AtWRKY63* is specific to abscisic acid-mediated stomatal closure and other signal transduction pathways [17]. The expression of *AtWRKY25*, *AtWRKY26* and *AtWRKY33* in *A. thaliana* was induced by ethylene under high temperature stress [19]. *TaWRKY44* plays a positive regulatory role in drought, salt and osmotic stress by activating the cell antioxidant system or the expression of related genes [7]. Overexpression of *TaWRKY1* and *TaWRKY33* in the nucleus of wheat can activate stress-related genes, increase germination rate, and promote root growth of *A. thaliana* in various stress environments [12]. *AtWRKY34* is up-regulated in cold and unique to pollen [7]. Under the conditions of an arid climate, *AtWRKY53* can promote metabolism, reduce the content of hydrogen peroxide, promote starch metabolism and regulate stomatal movement [20]. In addition, *WRKY* can also regulate the secondary metabolism of plants, such as the secondary metabolism of *artemisinin*. The *AaGSW1* gene activates the dual response of JA and ABA and is a positive regulator in the artemisinin biosynthesis pathway [21]. *GsWRKY20* in soybean can promote the expression of negative regulatory factors in ABA signal transduction and inhibit positive regulatory factors, which plays an important role in stomatal closure [22].

WRKY proteins are involved in plant growth and development, metabolism, senescence, biosynthesis and hormone signal regulation [7]. WRKY proteins are transcription factors with conserved WRKY domains, which play an important role as transcriptional repressors and activators [23]. The WRKY domain is composed of 4-strand β-sheets [1] and is composed of about 60 highly conserved amino acid residues [14]. It has one or two WRKY domains, including a conserved heptapeptide (WRKYGQK), coiled coil helix region and zinc finger motif. N-terminal heptapeptide WRKYGQK is the core sequence, and the C-terminal is composed of a C_2_H_2_ or C_2_HC zinc finger structure [3,24]. According to the number of WRKY domains, the type of coiled helix region and zinc finger motif, WRKY proteins can be divided into groups I, II and III [3,14,18]. Group I contains two WRKY domains and a C_2_H_2_ zinc finger motif and can be further divided into two subgroups: Ia has a C_2_H_2_ zinc finger motif and Ib has a C_2_HC zinc finger motif [14]; group II contains a WRKY domain and a C_2_H_2_ zinc finger motif and is further divided into five subgroups: IIa, IIb, IIc, IId, IIe [1,4,17]; group III consists of a WRKY domain and a C_2_HC zinc finger motif [3,14]. The WRKY domain is bound to the cis-acting element W-box, and the core sequence of the W-box (TTGACC/T) is necessary for binding to the WRKY. The target gene is regulated by cis elements, which reflects the conservation of the WRKY domain [1,14,17].

The analysis of flax WRKY transcription factors based on whole genome sequencing is rarely reported. Flax (*Linum usitatissimum* L.) is an important fiber and oil crop [25]. Due to its high-quality natural fiber, flax has many nutritional components and is rich in various phenolic compounds, vitamins and other substances. Flax can be used as textile, edible, medical and industrial raw materials, such as clothing fabrics, decorative fabrics, tablecloths, bedding and automotive supplies. Flax is one of the most widely distributed crops in the world, mainly distributed in China, Russia, France, Belgium, Poland, Egypt, the Czech Republic, the Netherlands, Britain, Spain and Austria. Oil flax is mainly distributed in India, Canada, China and America. China is the country with the largest flax planting, followed by Russia. Flax is an economically important crop and has a long cultivation history in China [18,26]. With the development of high-throughput sequencing technology, the related research direction is mainly sequencing the genomes of species, including rice (*Oryza sativa* L.), wheat (*Triticum aestivum* L.) and grape (*Vitis vinifera* L.), in addition to *A. thaliana* [26]. Genome-wide identification of *WRKY* genes has been carried out in many plant species [2]. In the model species, 128 *WRKY* genes have been identified, and 74 *WRKY* genes have been identified in *A. thaliana* [27]. In monocotyledonous plants, 136 *WRKY* genes were identified in maize (*Zea mays* L.) [28], 109 *WRKY* genes in rice (*Oryza sativa* L.) [29], 95 *WRKY* genes in palm oil (*Elaeis guineensis*) [30] and 121 *WRKY* genes in moso bamboo (*Phyllostachys edulis)* [31]. In dicotyledonous plants, 80 *WRKY* genes were identified in grape (*Vitis vinifera* L.) [32] and 145 *WRKY* genes were identified in Chinese cabbage (*Brassica rapa* ssp.) [33]. There are 104 *WRKY* genes in poplar (*Populus* L.) [34], 45 *WRKY* genes in *Eucommia ulmoides* [12], 95 *WRKY* genes in carrot (*Daucus carota* L.) [35], 76 *WRKY* genes in lettuce (*Lactuca sativa* L.) [12], 182 *WRKY* genes in soybean (*Glycine max* (Linn.) Merr.) [35], 55 *WRKY* genes in cucumber (*Cucumis sativus* L.) [36] and 81 *WRKY* genes in tomato (*Solanum lycopersicum*) [37]. In the flax genus, the genomes of three flax plants have been sequenced, and the flax data are relatively complete. Related research has predicted 43,384 protein-coding genes by whole genome-wide shotgun assembly, accounting for 93% of the published sequence tags (ESTs) in flax [38]. It can be applied to the in-depth study of evolutionary biology, which is of great significance to clarify the origin and evolution of species and accelerating the improvement of their economic value.

In this study, the whole genome WRKY transcription factors we identified from the sequenced genome and their phylogenetic relationship, gene structure, cis-acting elements, *WRKY* gene replication events, evolutionary relationship and phylogenetic classification were analyzed. This laid a foundation for further study on flax WRKY transcription factors.

## 2. Materials and Methods

### 2.1. Flax and Its Whole Genome Sequence

The genome information of flax (*Linum usitatissimum*) was downloaded from Phytozome, including: cds.fa; gene.gff3; protein.fa; transcript.fa and other files (https://phytozome.jgi.doe.gov/pz/portal.html) (accessed on 15 May 2020).

### 2.2. Identification of Genome-Wide WRKY Gene Family

The WRKY domain information was downloaded from Pfam (PF03106; http://pfam.xfam.org/family/PF03106) (accessed on 16 May 2020). The WRKY.hmm file downloaded by the hmmsearch program was used to screen the WRKY family genes in the flax genome-wide protein database, and the protein sequence with E-value < 1.2 × 10^−28^ was obtained. The known *A. thaliana* WRKY sequence was exploded, and a fasta file was established (WRKY.fasta) [6]. The hmm model Lusitatissimum-WRKY.hmm of the hmmalign.sto file was established by using hmmalign and hmmbuild programs. All flax protein sequence information was re-searched and the flax protein sequence was obtained. Lusitatissimum-WRKY.fasta with E-value < 0.01 was obtained. Subsequently, the domain was manually confirmed in SMART (http://smart.embl.de/) (accessed on 16 May 2020), NCBI CDD (https://www.ncbi.nlm.nih.gov/cdd/) (accessed on 16 May 2020) and Pfam (http://pfam.xfam.org/) (accessed on 16 May 2020), and the sequence with severe WRKY domain deletion was excluded in ExPASy (http://web.expasy.org/protparam/) (accessed on 15 May 2020) to obtain the final flax genome-wide *WRKY* gene family information database (Table S1).

### 2.3. Sequential Analysis

The obtained flax *WRKY* gene family database was classified and analyzed. Multiple alignment of WRKY protein sequences was performed by DNAMAN 6.0 software, and the protein sequence of the WRKY domain was identified and analyzed. According to the number of WRKY structures, WRKY was divided into 1R-WRKY, 2R-WRKY. MEGA was used to compare several sequences of WRKY flax gene family proteins, and the DNA binding domain of WRKY was identified. MEME (http://meme.nbcr.net/meme/intro.html) (accessed on 21 May 2020) was used to study the conservative region of flax WRKY [18,39].

### 2.4. Establishment of Phylogenetic Tree

Jalview manual correction was performed on a plurality of comparison results [40]. MEGA7 was used to generate the phylogenetic tree. The neighbor joining method, Whelan and Goldman + freq model were used to generate the phylogenetic tree of flax *WRKY* family genes.

### 2.5. Motif Analysis

The conserved sequence of *LuWRKYs* was obtained by the MEME program, using the following parameters: the maximum number of motifs was 15 motifs [6], and the optimal length was set to 10–100 according to previous experiments and surveys.

## 3. Results

### 3.1. Identification of WRKY Gene

We used HMMER to search for the WRKY domain (PF03106) in the whole flax genome and selected the protein sequence with E < 1.2 × 10^−28^ to construct a flax-specific WRKY.hmm. On this basis, the whole genome of flax was searched twice, the genes with E < 0.01 were screened out and 110 initial *WRKY* gene family data were obtained. After verification and analysis by Pfam, NCBI-CDD and SMART databases, 105 *WRKY* genes were obtained after removing some missing structural genes, including 26 in group I, 68 in group II, 8 in group III and 3 in group UN (Table 1). In addition, we identified 74 *WRKY* genes in *A. thaliana*, 145 *WRKY* genes in *B. rapa* and 86 *WRKY* genes in grapes by the same method in the reference species [27]. Through NCBI-CDD and Pfam detection, it was found that *Lus10006368* and ZAP1, *Lus10038426* and MEE24, *Lus10016282*, *Lus10012030* and *Lus10012027* are similar to MAPKKK11 or MEKK4.

*AtWRKY1*, *2*, *6*, *7*, *9*, *11*, *13*, *19*, *20*, *22*, *27*, *22*, *27*, *32*, *33*, *35*, *39*, *40*, *41*, *42*, *48*, *49*, *50*, *51*, *55*, *56*, *57*, *62*, *65*, *65*, *70*, *71*, *72*, *74*, *75* had high similarity and had weak similarity to A. thaliana *AtWRKY3* and *AtWRKY4*. The results showed that most flax *WRKY* gene families belong to group IIc and Ib. In group IIc, the zinc finger motif structure belongs to the Plant_zn_clust type, which belongs to the C_2_H_2_ zinc finger motif GCM1 family, but only 10 genes have a Plant_zn_clust domain, and only one WRKY domain is connected to the C terminal. In group IIc, 15 of them have coiled coil domains, and 2 of them have coiled coil domains, Plant_zn_clust and WRKY domains at the same time. There are 26 in group I, and they all have only two WRKY domains. There are three in group UN, and there are three WRKY domains (Table S1).

The flax *WRKY* gene family is complicated. *Lus10026409* and *Lus10022278* have a WRKY domain, but they are highly similar to *A. thaliana ATWRKY33. Lus10036401* and *Lus10007906* also have one WRKY domain, and have higher similarity to *ATWRKY56*, which may be related to the deletion of some *WRKYs*, so they are classified into group I. *Lus10001062*, *Lus10023099*, *Lus10001902*, *Lus10039331*, *Lus10032372*, *Lus10003894*, *Lus10034245* and *Lus10029022* have only one WRKY domain, but have high similarity to *AtWRKY41* or *AtWRKY55*, so they are classified to group III. *Lus10012678* has two WRKY domains, but it is highly similar to *AtWRKY13*, so it is placed into group I (Table S1).

### 3.2. Variation Analysis of Conserved Heptapeptide Sequence in WRKY Domain

**Figure 1:**
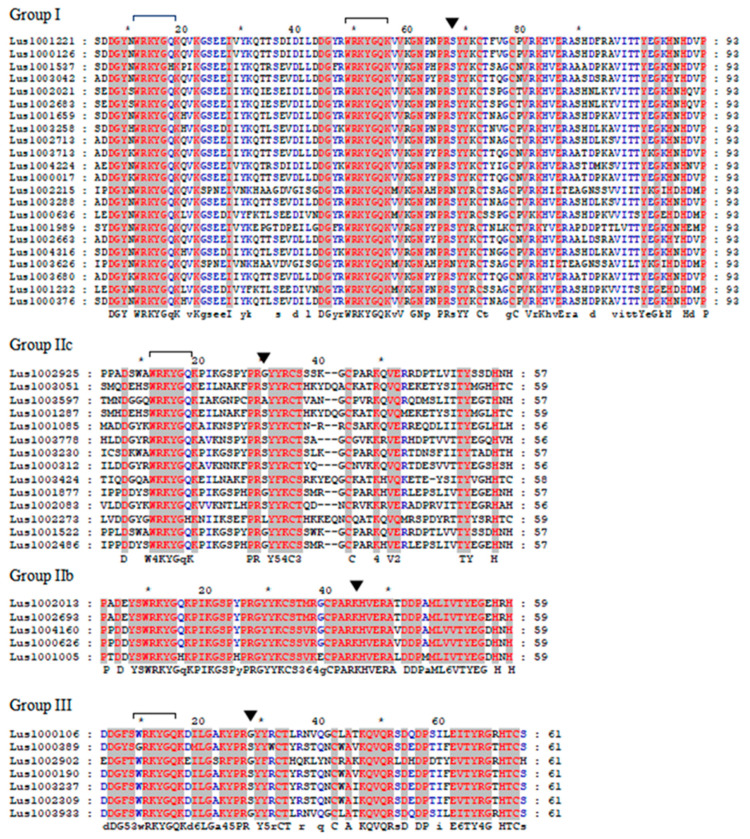
Variants of the conserved heptapeptide sequence of the WRKY domain in flax. Note: The WRKYGQK sequence, which is different from the conserved amino acid, is shown in red.

Multiple sequence alignment of the flax WRKY domain revealed that the heptapeptide of the conserved WRKY domain was WRKYGQK (Figure 1). Groups such as WRKYGQK, WRKYGKK, WRKYGHK, WRKYDQK, GRKYGQK and other groups (Figure 1) were also found, and these groups were further divided into Ia, Ib, IIc, IId, IIe, III and unclassified group UN. The majority of the WRKY domain sequences are WRKYGQK. The conserved domain of flax WRKY screened by HMMER was about 60 amino acids. There are two WRKYGQKs in group I, and the sequence information of heptapeptide is conservative after the second WRKY domain. Unlike other groups, the PRS (N) YK is S (N) amino acid. In the PRG (S/A/L) YK of group IIc, the S amino acid is relatively nonconserved. After PRKY, the conserved ARKH sequence in group IIb is similar to the PRG (S/A/L) YK region in group IIc.

**Figure 2:**
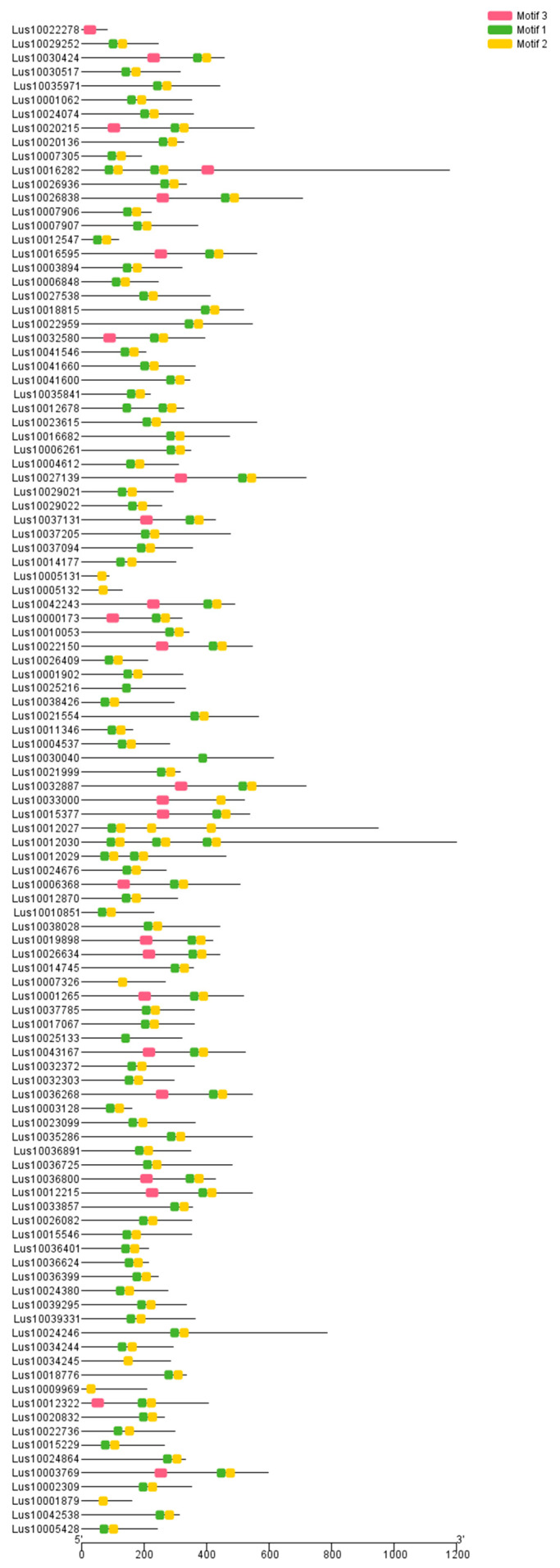

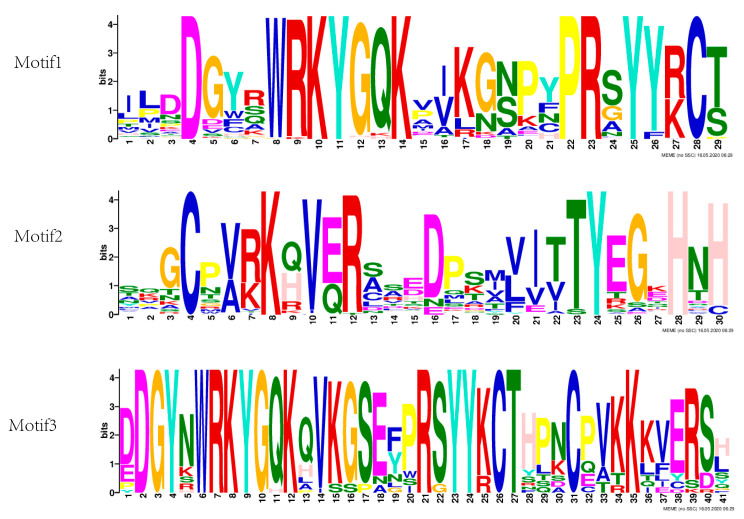
Conservative sequence structure analysis of flax *WRKY* family genes. Note: The relative size of the letters indicates their frequency in the sequence. The height of each letter is proportional to the frequency of the corresponding base at this position, often in bits.

The screened flax *WRKY* gene was introduced into the MEME website for domain detection and three groups of motifs were obtained (Figure 2). Genes in the same flax group have similar motifs. Most flax genes have two or three motifs at the same time, while only a few of them are single motifs.

**Figure 3:**
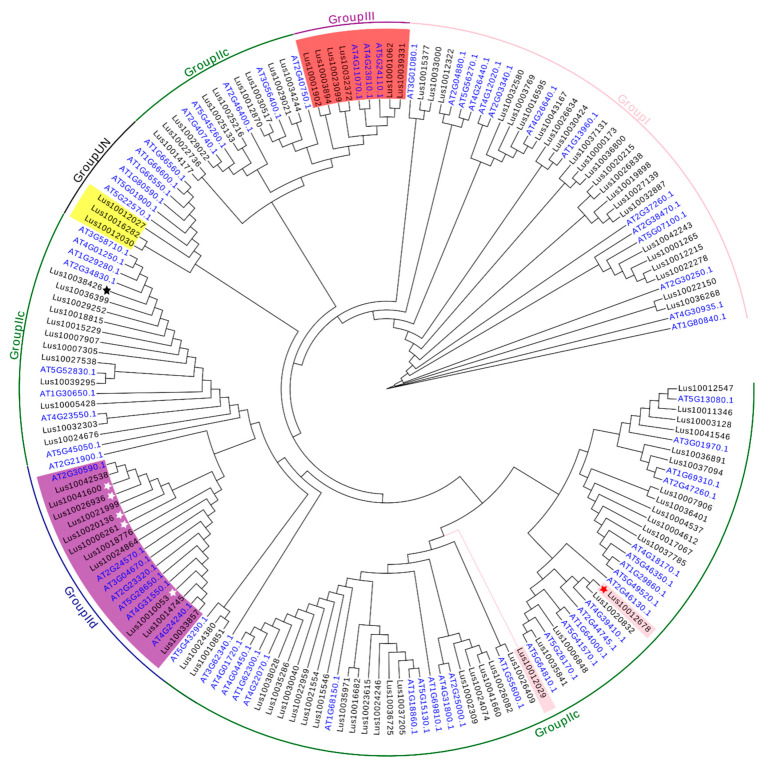
Phylogenetic tree of *WRKY* family genes in *flax.* Note: In the figure the black font is the *WRKY* gene of flax, and the blue font is the *WRKY* gene of *A. thaliana*. Each group/subgroup is displayed in different colors. The white star represents the flax gene of group IId, the red star represents the flax gene of group IIe, and the black star represents the flax gene with two WRKY domains in group IIc.

Combined with the conserved domain of the *A. thaliana WRKY* gene, the phylogenetic tree was constructed. The phylogenetic tree analysis aimed at revealing the evolutionary relationship between the flax gene and *A. thaliana WRKY* gene. According to the characteristics of the *WRKY* conserved domain, the *WRKY* gene families of flax and *A. thaliana* can be divided into three groups (Figure 2). It can be seen that both motif1 and motif3 have a typical sequence, WRKYGQK, with a complete WRKY conserved domain. There are 26 in the group I, most of which had C_2_H_2_ zinc finger motif and belong to subgroup Ia. Group II can be divided into three subgroups, including 68 (62.4%) *WRKY* genes. The *WRKY* gene closely related to IIc is clustered in groups IId and IIe, so there is a close evolutionary relationship. Group III contains eight *WRKY* genes, which appear in the recent era according to their position in the phylogenetic tree. The remaining three *WRKY* genes could not be divided into three major *WRKY* groups (Figure 3). Among all groups, the proportions of *WRKY* genes in groups I and II are the largest proportions.

## 4. Discussions

WRKY transcription factors play an important role in plant growth and development, signal transduction and metabolism. At the same time, WRKY is also involved in regulating the response of plants to various biotic and abiotic stresses [7]. With the development of high-throughput sequencing technology, WRKY family members have been identified at the whole genome level in many plants [2,26]. In this paper, a total of 105 *WRKY* gene sequences were screened and identified in flax. Due to the complexity of the WRKY family in flax, it was divided into four groups, groups I, II, III and UN. The number of genes corresponding to each group was 26, 68, 8 and 3, respectively, which could be subdivided into 8 subgroups, but flax was only distributed in 6 subgroups. Compared with the model plant *A. thaliana*, it was found that many genes in flax were highly similar to those in *A. thaliana*. The *WRKY* gene of flax was detected by domain detection, and three motifs were obtained, among which motif1 and motif3 had complete WRKY conserved domains. The *WRKY* gene of flax was combined with the conserved domain of the *A. thaliana WRKY* gene, and the phylogenetic tree of the flax gene was constructed to reveal the evolutionary relationship between the flax and *A. thaliana WRKY* genes.

WRKY transcription factors play an important role in regulating plant development and various stress responses [5]. *WRKY* genes plays an important role in responding to abiotic stresses, such as drought, salt, heat and osmotic. The corresponding *WRKY* genes in flax were studied as follows. *AtWRKY15* (*Lus10006261*, *Lus10041600*), *AtWRKY33* (*Lus10001265*, *Lus1001221 5*, *Lus10042243*, *Lus10026409*) and *AtWRKY40* (*Lus10002309*, *Lus10024074*, *Lus10026082*) were expressed under saline–alkaline stress [41]. In flax, the WRKY transcription factor gene *Lus10003894* was expressed only under alkaline stress, and *Lus10021554* and *Lus10022959* were expressed in neutral salt solution [26]. Under polyethylene glycol (PEG) treatment, the expression of *WRKY40* in flax seedlings increased, which helped flax plants to decrease the adverse effects of drought stress [41]. *A. thaliana AtWRKY2* homologous genes *Lus10027139* and *Lus10032887* are mainly expressed in the stems of flax and are highly expressed during flax fiber development. In addition, these genes can also regulate pollen formation and seed germination. The expression of homologous genes *Lus10020832* and *Lus10012678* of *AtWRKY13* in *A. thaliana* was positively correlated with the fiber content of flax, which had a positive regulatory effect on the fiber content of flax, however, the expression of *AtWRKY49* homologous gene *Lus10024380* in *A. thaliana* was negatively correlated with fiber content, which had a negative regulation effect [41]. The orthologous genes of *AtWRKY46* (*Lus10012870*, *Lus10025133*, *Lus10025216*), *AtWRKY54* (*Lus10 012870*, *Lus10025133*, *Lus10025216*) and *AtWRKY70* (*Lus10030517*) are related to the osmotic resistance of flax. The *Lus10001265*, *Lus10002309*, *Lus10012215*, *Lus10012870*, *Lus100240 74*, *Lus10026082*, *Lus10026409* and *Lus10043167* genes in flax were mainly in the subspecies [41]. It was found that when flax grew under unbalanced nutrient conditions, *WRKY33*, *WRKY40* and *WRKY70* were significantly expressed, and it was considered that *WRKY* family genes are also involved in the nutritional stress response of flax [42].

*WRKY* genes play an important role in plant growth and hormone regulation. *WRKY* genes have different regulatory effects on the growth and development of plants. Different genes have different regulatory effects, and one gene may also have different regulatory functions. For example, *WRKY75* is a positive regulator of leaf senescence, and *WRKY70* is a negative regulator of developmental senescence [1,2]. Salt stress or osmotic stress severely inhibits the development of lateral roots. *WRKY46* plays an important role in the development of lateral roots. When it is missing, the development of lateral roots can be significantly inhibited. When *WRKY46* is overexpressed, it can promote the development of lateral roots. This gene can help feed-forward inhibition to depend on lateral root inhibition by regulating the balance between ABA and IAA homeostasis [5]. In addition, *WKRY46* can regulate a group of genes that regulate cell osmotic protection and oxidative detoxification under drought and salt stress and also regulate stomatal opening by participating in the regulating of starch metabolism in guard cells [43]. Genes in *A. thaliana* (*AtWRKY18*, *AtWRKY40* and *AtWRKY60*) [10] and rice (*OsWRKY11*, *OsWRKY71*, *OsWRKY72* and *OsWRKY77*) [11] can induce abscisic acid. Therefore, it is necessary to further strengthen the research on the *WRKY* gene in flax growth and development, stress response and hormone regulation response, so as to clarify the various pathways and physiological processes involved in the flax *WRKY* gene and target gene in flax and find the corresponding candidate genes. The study of the flax *WKRY* gene family is still a hot spot in flax research.

## 5. Conclusions

WRKY is one of the largest transcription factor families in plants and plays an important role in plant growth, development, senescence and biotic and abiotic stress. *WRKY* regulates the processes of plants by forming components of signal networks. The *WRKY* gene is involved in regulating important plant processes through inhibition or activation. Flax WRKY transcription factors play an important role in the regulation of osmotic stress tolerance in plants. In this study, the members of the flax *WRKY* gene family were searched on a genome-wide basis. A total of 105 *WRKY* genes were obtained from the *WRKY* gene library of flax, and the *LuWRKYs* were divided into four groups. There were 26 *WRKY* genes in group I, 68 *WRKY* genes in group II, 8 *WRKY* genes in group III and 3 *WRKY* genes in group UN. These groups can then be subdivided into eight subgroups. Through WRKY domain analysis, it was found that multiple *WRKYs* had high similarity, and the *WRKY* genes in flax had high similarity to the genes in *A. thaliana*. Three motifs were obtained from the domain detection of the selected *WRKY* gene. In this paper, the *WRKY* genes in flax were described comprehensively, providing a theoretical basis and significance for the further study of the role of WRKY genes in flax in growth, development and response to adversity.

## Figures and Tables

**Table 1:** Number of genes encoding WRKY proteins in flax and other plants.

Name	*L. usitatissimum*	*A. thaliana*	*B. rapa*	*V. vinifera*
Group Ⅰ	26	13	31	12
Group Ⅱa	0	3	7	3
Group Ⅱb	0	8	17	8
Group Ⅱc	62	17	38	15
Group Ⅱd	1	10	13	7
Group Ⅱe	5	8	14	6
Group Ⅲ	8	13	23	6
Group UN	3	0	0	2
Total	105	72	143	59

## Data Availability

No new data were created.

## Supplementary Materials

The following supporting information can be downloaded at: https://www.mdpi.com/article/10.3390/life13061258/s1. Table S1: Information of flax WRKY gene family.
